# Impact of COVID-19 Pandemic on Health and Life Emergencies Resulting from Illness Cases and Injuries—A Preliminary Study

**DOI:** 10.3390/jcm13123552

**Published:** 2024-06-18

**Authors:** Krzysztof Marek Mitura, Daniel Celiński, Jadwiga Snarska, Sławomir Dariusz Szajda

**Affiliations:** 1 Independent Public Health Care Center RM-MEDITRANS Emergency Station and Sanitary Transport in Siedlce, 08-110 Siedlce, Poland; 2 Department of Emergency Medical Service, Medical University of Warsaw, 02-097 Warsaw, Poland; 3 Department of Surgery, Collegium Medicum, University of Warmia and Mazury in Olsztyn, 10-719 Olsztyn, Poland; 4 Department of Emergency Medical Service, Collegium Medicum, University of Warmia and Mazury in Olsztyn, 10-719 Olsztyn, Poland

**Keywords:** health and life emergency, SARS-CoV-2, COVID-19, pandemic, illness case, injury

## Abstract

**Background:** Despite organised efforts, the COVID-19 pandemic had a significant impact on the health status of the population and health services including the emergency medical system. The objective of the study was to investigate, based on the Emergency Medical Teams’ (EMT) interventions, the impact of the COVID-19 pandemic on health and life emergencies resulting from illness cases and injuries of Polish females and males. **Material and Methods:** The data under analysis concern EMT interventions carried out in central and eastern Poland from 1 January 2017 to 31 December 2022 (*n* = 226,038). The study used descriptive statistics, the Mann–Whitney U Test, and the Chi-square test. **Results:** A significant increase was observed in the proportion of EMT interventions (*p* < 0.001) to patients with illness cases (80.30% vs. 83.17%) and a decrease in interventions to patients with injuries (19.70% vs. 16.83%) during the pandemic as compared to the pre-pandemic period. As for illness cases, the patients’ ages during both periods were similar (Me = 66.00 vs. 66.00, *p* = 0.071). On the other hand, during the pandemic, injuries mainly affected elderly patients as compared to the pre-pandemic period (Me = 50.00 vs. 47.00, *p* < 0.001). The increase in the proportion of EMT interventions to patients with illness cases and the decrease in patients with injuries during the pandemic, as compared to the pre-pandemic period, concerned the area of intervention, patient’s sex, and age. During the pandemic period, a significantly lower proportion of patients transported to the hospital (*p* < 0.001) and an increase in the proportion of patients left at the place of call (*p* < 0.001) were noted. **Conclusions:** The restrictions aimed at preventing the spread of the SARS-CoV-2 virus contributed to a reduced number of injuries without, however, reducing the number of illness cases. During the pandemic, the elderly were affected by injuries. The study indicates the need for further in-depth analyses to prepare the pre-hospital care system in Poland for the occurrence of other or similar emergencies.

## 1. Introduction

The emergency medical system aims to provide the quickest possible, most effective, and professional assistance to people in a health or life emergency. Health and life emergencies refer to situations in which serious disorders of the human body, bodily harm, or even death are expected to occur suddenly or within a short period of time. They require the rapid implementation of rescue measures, which, especially in the pre-hospital setting, should represent consecutive, complementary stages of providing assistance, forming links in the rescue chain. Starting from emergency notification, through interventions carried out by event witnesses and emergency medical teams, and ending with hospital treatment, the rescue chain allows assistance to be provided effectively and underlies the most effective emergency medical systems [1,2].

The causes of health and life emergencies include illness cases and injuries that can be distinguished on the basis of diagnoses made by qualified and authorised medical personnel. Diagnoses are made on the basis of uniform codification that enables reliable reporting and comparison of medical cases encountered both in the pre-hospital setting and throughout the healthcare system [3,4]. This is precisely the role served by the International Statistical Classification of Diseases and Related Health Problems International Classification of Diseases—ICD [5].

The distribution of illness cases and injuries can be presented using the Disability-Adjusted Life-Years (DALY) index, a measure of disability-adjusted life expectancy consisting of disease burden, premature mortality and the incidence and severity of ill health. In 2017, globally, for all age groups and all DALY causes, the largest proportion was represented by non-communicable diseases (62.0%), followed by infectious, maternal, neonatal and nutritional diseases (27.9%) and injuries (10.1%) [6].

However, based on the Global Burden of Diseases, Injuries and Risk Factors Study, non-communicable diseases caused 73.4% of deaths in 2017. Infectious, maternal, neonatal and nutritional causes accounted for 18.6%, and injuries for 8.0% of deaths [7]. 

The emergence of the SARS-CoV-2 virus (severe acute respiratory syndrome coronavirus 2) in 2019, followed by its rapid spread, resulted in the pandemic outbreak. The pandemic and the restrictions introduced involved keeping social distance, staying at home, studying, and working remotely to limit the spread of the virus, which affected social life and various aspects of public health. During the COVID-19 pandemic, there was an increase in the impact of stressors on people’s lives and health related to reduced income, job loss as well as mental illnesses and the use of psychoactive substances, which are known risk factors for the occurrence of injuries [8]. In addition, post-injury patients negatively perceived the impact of the COVID-19 pandemic on their recovery and access to the healthcare system [9].

In Europe, the pandemic resulted in a reduction in the number of hospital admissions for ischaemic heart diseases, cerebrovascular diseases, and heart failure, as well as urgent and elective cardiac surgeries and an increase in cases of cardiac arrests [10]. Mortality rates were considerably higher during the pandemic, as compared to the pre-pandemic period, for ischaemic heart diseases and cerebrovascular diseases [10].

A British study indicated that between April and August 2020, more than 30,000 fewer patients started treatment for cancer than in the same period of the previous year [11]. Another study suggests an increase, within a period of up to five years after diagnosis, in the number of additional deaths caused by breast, lung, oesophageal and colorectal cancers as a result of delayed diagnosis due to the COVID-19 pandemic [12]. In Poland, at the beginning of the COVID-19 pandemic, there was a significantly lower number of breast and cervical cancer screening tests performed on women [13].

A number of studies in numerous countries indicate that since the onset of the COVID-19 pandemic, there has been a significant increase in the prevalence of symptoms associated with mental ill health and mental disorders [14] and an increasing demand for mental health medical services [15].

The impact of the COVID-19 pandemic on the provision of health services and the characteristics of patients receiving medical services is apparent. The adverse impact of the SARS-CoV-2 virus on the health of the population particularly concerned people with chronic diseases and was mainly due to the difficulty for a sick person to obtain medical services at a time when the focus was on the COVID-19 pandemic [16,17].

The study’s objective was to investigate the impact of the COVID-19 pandemic on health and life emergencies resulting from illness and injuries and the functioning of pre-hospital care in emergency situations in central and eastern Poland.

## 2. Materials and Methods

### 2.1. Study Design

The study was carried out on a 7350 km^2^ area of central and eastern Poland, with a population of approximately 532,000 [18]. A retrospective analysis covered interventions carried out by Emergency Medical Teams (EMT) from 1 January 2017 to 31 December 2022.

In order to make comparisons, the time interval under study was divided into two periods: the pre-pandemic period (from 1 January 2017 to 31 December 2019) and the COVID-19 pandemic period (from 1 January 2020 to 31 December 2022). The start of the pandemic period was set to be 1 January 2020 because of the difficulty in clearly identifying the exact date due to the rapidity of the virus spread and the initial difficulties in diagnosing infected patients. The COVID-19 disease was first diagnosed at the end of 2019 in the city of Wuhan, China, and as early as 11 March 2020, the World Health Organization (WHO) declared a COVID-19 pandemic. In Poland, the first official case of COVID-19 infection was noted on 4 March 2020. On 14 March 2020, a state of epidemic emergency was declared, while on 20 March 2020, a state of epidemic was announced.

### 2.2. Research Data

The data on illness cases (*n* = 184,650) and injuries (*n* = 41,388) were acquired using the State Emergency Medical Service Command Support System, which is an electronic register of EMT interventions in Poland. The data used in the study included the age, sex, the area of EMT intervention (urban, rural), time of intervention, the diagnosis made according to the International Classification of Diseases (ICD-10), and the result of intervention. Based on the ICD-10 codification, all the diagnoses made were categorised into illness cases and injuries. The analyses that were conducted only concerned the pre-hospital management of patients.

The study, due to its nature and the data it analysed, required no approval from the Research Ethics Committee, as it required no human participation and, thus, no informed consent to participate in the study.

### 2.3. Statistical Analysis

The data under analysis were stored in a Microsoft Excel MS Office 2021 database for Windows 11 software. For statistical analyses and visualisation of their results, the study used R ver. 4.3.1 [19] with the RStudio ver. 2023.09.1.494 environment [20] and a *tidyverse* package set ver. 2.0.0 [21].

The Kolmogorov-Smirnov test was used to assess the normality of distribution. In order to compare the patients’ ages (in years) between the pre-pandemic and pandemic periods, the Mann–Whitney non-parametric U test as well as descriptive statistics, namely, the median (Me) and interquartile range (IQR), were applied. The most popular methods were used to investigate the relationships between the qualitative variables, the Chi-squared test (χ^2^) was applied, and in their description, the numerosity (*n*) and percentage (%) were used. Statistically significant results were recognised at *p* < 0.050.

## 3. Results

From 1 January 2017 to 31 December 2022, EMTs operating in central and eastern Poland carried out 226,038 interventions to patients, of which 81.69% (*n* = 184,650) were interventions to patients with illness cases and 18.31% to patients with injuries (*n* = 41,388).

**Figure 1:**
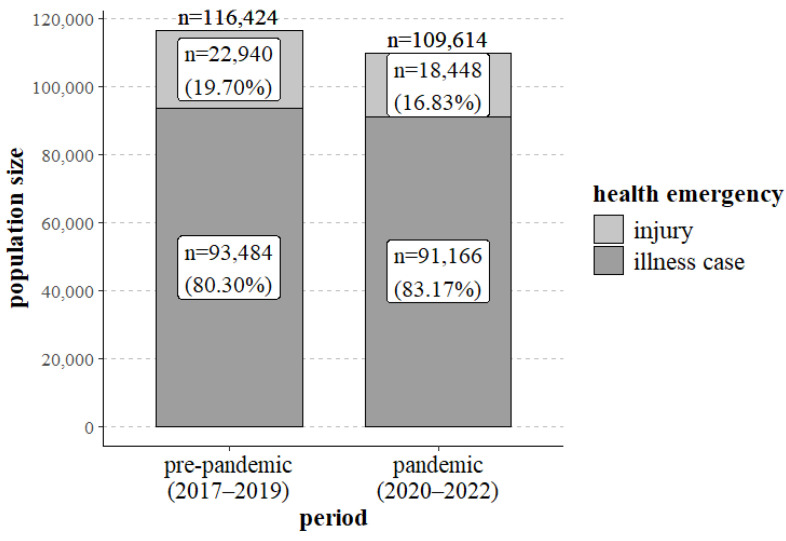
EMT interventions for illness cases and injuries in the pre-pandemic period and during the COVID-19 pandemic.

When comparing the pre-pandemic period (2017–2019) with the pandemic period (2020–2022), significant differences were noted in the number of EMT interventions for patients with illness cases and injuries (*p* < 0.001). As shown in Figure 1 and Table 1, during the pandemic period, the proportion of interventions to patients with illness cases increased significantly (80.30% vs. 83.17%), whereas the proportion of interventions to patients with injuries decreased (19.70% vs. 16.83%) (Figure 1, Table 1).

**Figure 2:**
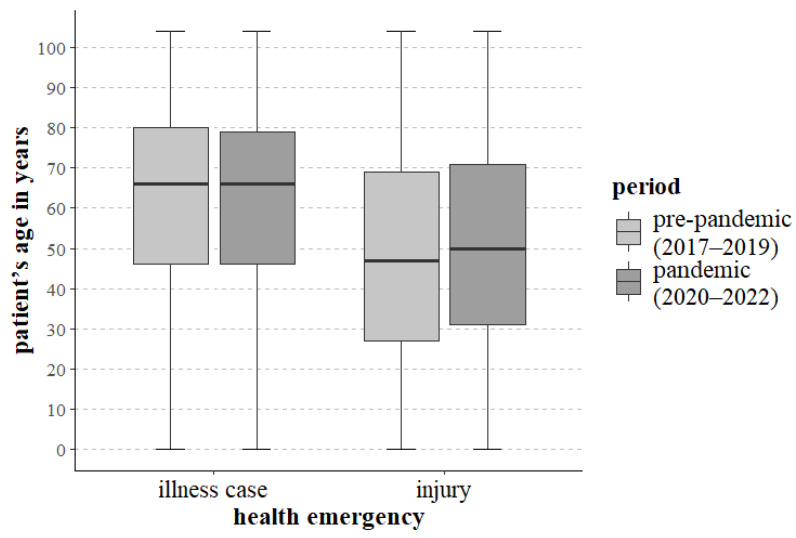
Characteristics of the age of patients with health and life emergencies resulting from illness cases and injuries in the pre-pandemic period and during the COVID-19 pandemic.

Regardless of the reason for intervention and the period under analysis, the age range of patients to whom EMTs were dispatched ranged from birth to 104 years. As regards interventions for illness cases, no significant statistical differences regarding patients’ ages were noted between the periods under study (*p* = 0.071). With regard to illness cases, the median ages were similar during the two periods. Statistically significant differences (*p* < 0.001) between the studied periods were, however, noted for the age of patients affected by injuries. During the pandemic period, injuries affected older patients more than during the pre-pandemic period. The data covering the patients’ ages, as well as the period and type of potential health and life emergency, are provided in Figure 2 and in Table 2.

Significant differences between the periods under analysis were noted for the number of calls for EMT interventions to patients with illness cases and injuries, depending on the area of intervention: urban (*p* < 0.001) and rural (*p* < 0.001), patient’s sex—male (*p* < 0.001), female (*p* < 0.001), and patient’s age—age group of up to and including 14 years (*p* < 0.001), 15–29 (*p* < 0.001), 30–49 (*p* < 0.001), 50–69 (*p* < 0.001), 70 and more years (*p* < 0.001). Compared to the pre-COVID-19 pandemic period, the highest decrease in the proportion of injuries during the pandemic was noted in the age group of up to and including 14 years (a reduction of 10.11%), whereas the lowest one was noted in the age group of 70 and more years (a reduction of 0.96%). Thus, during the pandemic, there was an analogous increase in the proportion of illness cases depending on the increasing age of the patients (Table 3).

In addition, significant differences were noted for calls for EMT interventions to patients with illness cases and injuries, resulting in patients being transported to the hospital (*p* < 0.001) or left at the place of call (*p* < 0.001). As for the two types of health and life emergency, a decrease in the proportion of patients transported to the hospital and an increase in the proportion of patients left at the place of call was noted during the pandemic period (Table 4).

Regarding EMT interventions for illness cases and injuries during which the patient was pronounced dead, no significant differences were noted between the periods under comparison (*p* = 0.510) (Table 5).

## 4. Discussion

After the so-called Spanish Flu pandemic, the COVID-19 pandemic was the greatest public health challenge faced by the modern world. The SARS-CoV-2 virus brought about changes in the organisation of the health care system and the characteristics of patients receiving health care services, including at the pre-hospital stage. Although the restrictions introduced limited the spread of the COVID-19 pandemic, they became factors affecting various aspects of social and economic life.

The current study indicates that during the COVID-19 pandemic (*n* = 109,614), as compared to the pre-pandemic period (*n* = 116,424), there was a reduction in the number of medical services provided by EMTs (Figure 1, Table 1). From a public health perspective, this may suggest that patients did not use the emergency medical system with the same frequency as they had before the pandemic period. This may be due to patients’ fear of being infected with the virus during contact with EMTs and patients possibly seeking medical help directly at healthcare facilities or via online consultation. A reduction in the number of EMT interventions was also observed in Germany, particularly during periods when strict precautions were in place to prevent the spread of the SARS-CoV-2 virus [22]. A lower number of EMT interventions and emergency calls, particularly in the initial period of the pandemic, were observed throughout the United States [23]. It may appear that fewer EMT interventions during the pandemic period could have a positive, disburdening effect on emergency medical systems, as long as people whose health needs do not require health care services seek other medical care options [23].

Based on the authors’ own research, during the pandemic, an increase was observed in the proportion of EMT interventions to patients with illness cases (from 80.30% to 83.17%) and fewer interventions to patients with injuries (from 19.70% to 16.83%) (Table 1). Orders to stay at home and the introduction of remote working and studying translated into less frequent use of motor vehicles, fewer risky activities, and fewer sports, thus reducing the possibility of receiving injuries. Dicker et al. noted that in New Zealand, the number of EMT interventions for patients with injuries significantly decreased, as did the number of traffic accidents [24]. A similar trend was observed by Lerner et al. in the United States, where the number of EMT calls to patients with injuries fell from 18.43% in the pre-pandemic period to 15.27% as compared to the initial period of the pandemic [23]. On the other hand, in Tyrol, Austria, an increase of 18.7% in the number of emergency calls due to respiratory disorders and a decrease of 26.4% in the number of emergency calls due to traffic incidents, as compared to the years 2017–2019, were observed [25]. At the trauma centre in Breda, the Netherlands, there was a 32% reduction in the number of injuries in the first months of the pandemic, as compared to the years preceding the pandemic (2018–2019), with the largest reduction in the number of sports-related injuries. Moreover, injuries due to falls from height and those received when performing work were observed more frequently [26].

The current research found that in the case of EMT interventions for patients with illness cases before and during the COVID-19 pandemic, the age of patients receiving emergency medical services was similar (*p* = 0.071). The median age during and before the pandemic period was 66.00 (Figure 2, Table 2). During the pandemic period, injuries affected older patients more than during the pre-pandemic period. The median age of patients with injuries during the pandemic period was 50.00 years, compared to 47.00 years for the pre-pandemic period (Figure 2, Table 2). A significant increase in the age of patients who suffered from an injury during the pandemic may be related to the restrictions introduced, including those on movement and ordering social distance, which mainly affected people of school and working age. A study involving patients hospitalised for injuries in Lubelskie voivodeship in Poland indicates that the median age of patients with injuries increased from 40 years in the pre-pandemic period to 46 years during the pandemic [27]. A Dutch study also observed that during the pandemic, the mean age of patients with injuries was higher and amounted to 48 years as compared to 42 years in 2019 and 43 years in 2018 [26].

The current study also found a significant reduction in the number of injuries during the pandemic as compared to the pre-pandemic period, regardless of whether the event took place in a rural or an urban area. There was also a significant reduction in the number of injuries during the pandemic period for both females and males. In addition, significant reductions in the number of injuries during the pandemic were observed in each of the analysed age groups (Table 3). At the same time, the study found that the younger the patients, the greater these reductions were. In the age group of up to and including 14 years, the proportion of injuries during the pandemic was 32.03%, compared to 42.14% in the pre-pandemic period, whereas injuries in patients aged 70 years and older accounted for 11.20% and 12.16%, respectively, of all EMT interventions (Table 3). During the pandemic, older patients required a higher proportion of interventions compared to younger patients. The decrease in the number of injuries during the COVID-19 pandemic can be attributed to the restrictions introduced to prevent the spread of the virus, as well as factors such as the patient’s sex and age group. These restrictions, as mentioned earlier, reduce the risk of injuries, especially in younger patients who are characterised by greater mobility and a tendency to engage in risky activities as compared to the elderly. Jojczuk et al. report that in 2020, compared to 2019, the proportion of patients with injuries admitted to hospitals in Lubelskie voivodeship (Poland) decreased in the age groups of 1–17 years (to 10.4% from 16.3%) and 18–45 years (to 39.5% from 40.4%), whereas it increased in the age ranges of 46–65 years (to 25.7% from 23.8%) and over 65 years (to 24.4% from 19.5%) [27]. In 2020, compared to the pre-pandemic period, in the Children’s Hospital in Montreal, Canada, a reduction in the number of injury-related visits to the emergency department was observed in all age groups, with the smallest decrease noted for children aged 2 to 5 years (35%), and the largest decrease for the 12- to 17-year-old group (83%) [28].

The current study showed that during the pandemic period, there was a decrease in the proportion of patients transported to the hospital due to illness cases (72.80% vs. 67.60%) and injuries (88.55% vs. 86.82%), with a simultaneous increase in the proportion of patients who were left at the place of call after having received medical attention, for both illness cases (27.20% vs. 32.40%) and injuries (11.45% vs. 13.18%) (Table 4). This may suggest patients’ fear of a possible SARS-CoV-2 virus infection in case of transport to and stay in hospital. Irrespective of the EMT intervention type (injury, illness case), Dicker et al. indicate an increase in the number of patients left at the place of call during the pandemic period (30.5% vs. 20.9%) [24]. Similar trends for the increase in the number of patients left at the place of call between 2019 and 2020 were observed in Israel (13.4% vs. 19.9%) [29] and Finland (36.9% vs. 39.9%) [30].

Based on the current analyses, no differences were observed in the two study periods covering EMT interventions for patients with illness cases or injuries in which a patient’s death occurred (*p* = 0.510) (Table 5). However, an American study indicated that during the first period of the pandemic, as compared to the pre-pandemic period, the number of deaths increased two times (from 1.49 to 2.77%) for all calls for EMT intervention [23]. An Italian study from the Lombardy region notes that during the COVID-19 epidemic outbreak in 2020, as compared to 2019, witnesses to the event were less inclined to undertake cardiac pulmonary resuscitation (20% vs. 31%) [31]. A meta-analysis by Lim et al. showed that the incidence and mortality for out-of-hospital cardiac arrest were higher during the COVID-19 pandemic compared to the pre-pandemic period [32]. On the other hand, Emigh B. et al. point out that SARS-CoV-2 virus infection in patients with injuries was associated with a five- to sixfold increase in the risk of death as compared to non-infected patients with injuries [8]. At the same time, according to the WHO, there was an increase in deaths worldwide in 2020 and 2021, amounting to 7.97% and 18.30%, respectively, as compared to what would have been expected if the pandemic had not occurred [33].

The current study’s major advantage is that it was conducted using data acquired from the nationwide, integrated State Emergency Medical Service Command Support System. However, further in-depth research is needed to analyse the impact of the COVID-19 pandemic on the health situations of individual patient groups and the functioning of the healthcare system.

This investigation is subject to several limitations that warrant consideration. The first limitation is that this is a retrospective study, and the study was conducted in a single centre. The study was conducted among patients who received pre-hospital diagnoses, and the specificity of EMS activities means that medical staff may not be able to identify all signs and symptoms of diseases and injuries.

## 5. Conclusions

This study indicates a number of significant changes with regard to health and life emergencies resulting from illness cases and injuries, which are caused by the COVID-19 pandemic. During the pandemic, the proportion of EMT interventions to illness cases increased, whereas the proportion of interventions to injuries decreased, with injuries affecting elderly patients to a greater extent than in the pre-pandemic period. It is fair to assume that the reduction in the number of injuries is linked to the introduction of restrictions on movement, isolation, and remote studying and working, which were aimed at preventing the spread of the SARS-CoV-2 virus. There is also a need for broader analyses of the causes and types of illness cases and injuries during the COVID-19 pandemic as compared to the pre-pandemic period.

A comparative analysis of the functioning of the Polish State Emergency Medical Service system in the periods before and during the COVID-19 pandemic can improve the performance of pre-hospital care and prepare it for other potential emergencies, which can have a positive impact on public health and the economy.

## Figures and Tables

**Table 1:** EMT interventions for illness cases and injuries in the pre-pandemic period and during the COVID-19 pandemic.

Health Emergency Type	Period		Test	
	Pre-Pandemic (2017–2019)	Pandemic (2020–2022)	χ^2^ (df)	*p*-Value
	*n* (%)	*n* (%)		
**Illness case**	93,484 (80.30)	91,166 (83.17)	311.75 (1)	***p* < 0.001**
**Injury**	22,940 (19.70)	18,448 (16.83)		
**Total**	**116,424**	**109,614**		

**Table 2:** Characteristics of the age of patients with health and life emergencies resulting from illness cases and injuries in the pre-pandemic period and during the COVID-19 pandemic.

	Health Emergency Type	Parameters	Period		Test	
			Pre-Pandemic (2017–2019)	Pandemic (2020–2022)	Mann–Whitney U	*p*-Value
**Age (years)**	**illness case**	***n***	**93,484**	**91,166**	W = 4,281,931,265	*p* = 0.071
		median (IQR)	66.00 (46.00–80.00)	66.00 (46.00–79.00)		
		range	0–104	0–104		
	**injury**	***n***	**22,940**	**18,448**	W = 200,396,593	***p* < 0.001**
		median (IQR)	47.00 (27.00–69.00)	50.00 (31.00–71.00)		
		range	0–104	0–104		

**Table 3:** The effects of the area of intervention, sex and age on health and life emergencies resulting from illness cases and injuries in the pre-pandemic period and during the COVID-19 pandemic.

Variables		Health Emergency Type	Period		Test	
			Pre-Pandemic (2017–2019)	Pandemic (2020–2022)	χ^2^ (df)	*p*-Value
			*n* (%)	*n* (%)		
**Area**	**urban**	illness case	31,378 (80.33)	30,508 (83.55)	131.93 (1)	***p* < 0.001**
		injury	7685 (19.67)	6008 (16.45)		
	**rural**	illness case	62,106 (80.28)	60,658 (82.98)	182.57 (1)	***p* < 0.001**
		injury	15,255 (19.72)	12,440 (17.02)		
**Sex**	**female**	illness case	46,218 (83.02)	45,125 (85.65)	141.87 (1)	***p* < 0.001**
		injury	9454 (16.98)	7559 (14.35)		
	**male**	illness case	47,266 (77.80)	46,041 (80.87)	168.83 (1)	***p* < 0.001**
		injury	13,486 (22.20)	10,889 (19.13)		
**Age group**	**0–14**	illness case	2893 (57.86)	3009 (67.97)	102.5 (1)	***p* < 0.001**
		injury	2107 (42.14)	1418 (32.03)		
	**15–29**	illness case	7165 (62.71)	5979 (67.25)	55.47 (1)	***p* < 0.001**
		injury	4260 (37.29)	2846 (32.25)		
	**30–49**	illness case	16,014 (74.32)	16,600 (77.68)	66.03 (1)	***p* < 0.001**
		injury	5532 (25.68)	4771 (22.32)		
	**50–69**	illness case	26,894 (83.19)	26,028 (85.47)	61.51 (1)	***p* < 0.001**
		injury	5433 (16.81)	4424 (14.53)		
	**70+**	illness case	40,518 (87.84)	39,550 (88.80)	20.09 (1)	***p* < 0.001**
		injury	5608 (12.16)	4989 (11.20)		

**Table 4:** The result of EMT interventions (patient transported to hospital or left at the place of call) for illness cases and injuries in the pre-pandemic period and during the COVID-19 pandemic.

Health Emergency Type	Result of Intervention	Period		Test	
		Pre-Pandemic (2017–2019)	Pandemic (2020–2022)	χ^2^ (df)	*p*-Value
		*n* (%)	*n* (%)		
**Illness case**	patient transported to the hospital	66,212 (72.80)	59,981 (67.60)	581.64 (1)	***p* < 0.001**
	patient left at the place of call	24,736 (27.20)	28,750 (32.40)		
**Injury**	patient transported to the hospital	20,194 (88.55)	15,912 (86.82)	28.57 (1)	***p* < 0.001**
	patient left at the place of call	2610 (11.45)	2416 (13.18)		

**Table 5:** Deaths due to illness cases and injuries in the pre-pandemic period and during the COVID-19 pandemic.

**Death**	**Health Emergency Type**	**Period**		**Test**	
		**Pre-Pandemic (2017–2019)**	**Pandemic (2020–2022)**	**χ^2^ (df)**	***p*-Value**
		***n* (%)**	***n* (%)**		
	**illness case**	2536 (94.91)	2435 (95.30)	0.43 (1)	*p* = 0.510
	**injury**	136 (5.09)	120 (4.70)		

## Data Availability

The dataset is available from the corresponding authors upon reasonable request and with permission from the Independent Public Health Care Center RM-MEDITRANS Emergency Station and Sanitary Transport in Siedlce, Poland.
